# Epigallocatechin-3-Gallate as a Novel Vaccine Adjuvant

**DOI:** 10.3389/fimmu.2021.769088

**Published:** 2021-11-12

**Authors:** Yucheol Cheong, Minjin Kim, Jina Ahn, Hana Oh, Jongkwan Lim, Wonil Chae, Seung Won Yang, Min Seok Kim, Ji Eun Yu, Sanguine Byun, Yo Han Jang, Baik Lin Seong

**Affiliations:** ^1^ Department of Biotechnology, College of Life Science and Biotechnology, Yonsei University, Seoul, South Korea; ^2^ Graduate Program in Biomaterials Science and Engineering, College of Life Science and Biotechnology, Yonsei University, Seoul, South Korea; ^3^ The Interdisciplinary Graduate Program in Integrative Biotechnology & Translational Medicine, Yonsei University, Incheon, South Korea; ^4^ Department of Biological Sciences and Biotechnology Major in Bio-Vaccine Engineering, Andong National University, Andong, South Korea; ^5^ Vaccine Industry Research Institute, Andong National University, Andong, South Korea; ^6^ Department of Microbiology, College of Medicine, Yonsei University, Seoul, South Korea; ^7^ Vaccine Innovative Technology ALliance (VITAL)-Korea, Yonsei University, Seoul, South Korea

**Keywords:** EGCG, adjuvant, IgG isotype switching, influenza, ADCC

## Abstract

Vaccine adjuvants from natural resources have been utilized for enhancing vaccine efficacy against infectious diseases. This study examined the potential use of catechins, polyphenolic materials derived from green tea, as adjuvants for subunit and inactivated vaccines. Previously, catechins have been documented to have irreversible virucidal function, with the possible applicability in the inactivated viral vaccine platform. In a mouse model, the coadministration of epigallocatechin-3-gallate (EGCG) with influenza hemagglutinin (HA) antigens induced high levels of neutralizing antibodies, comparable to that induced by alum, providing complete protection against the lethal challenge. Adjuvant effects were observed for all types of HA antigens, including recombinant full-length HA and HA1 globular domain, and egg-derived inactivated split influenza vaccines. The combination of alum and EGCG further increased neutralizing (NT) antibody titers with the corresponding hemagglutination inhibition (HI) titers, demonstrating a dose-sparing effect. Remarkably, EGCG induced immunoglobulin isotype switching from IgG1 to IgG2a (approximately >64–700 fold increase), exerting a more balanced T_H_1/T_H_2 response compared to alum. The upregulation of IgG2a correlated with significant enhancement of antibody-dependent cellular cytotoxicity (ADCC) function (approximately 14 fold increase), providing a potent effector-mediated protection in addition to NT and HI. As the first report on a novel class of vaccine adjuvants with built-in virucidal activities, the results of this study will help improve the efficacy and safety of vaccines for pandemic preparedness.

## Introduction

Classical vaccines composed of whole virions or bacteria, such as live attenuated or inactivated vaccines, have inherent immune-stimulating effects exerted by various components, including proteins, lipids, and nucleic acids. However, new generation vaccine platforms such as subunit vaccines or nucleic acid (e.g. DNA) vaccines are relatively less immunogenic and, thus, require appropriate adjuvants to enhance their immunogenicity (1, 2). Since most vaccines are used in healthy people for prophylactic purposes, both vaccines and adjuvants are required to demonstrate a high level of safety and tolerability. Many factors should be taken into account for the proper selection of adjuvants, including the vaccine type, desired immune response, administration route, and target population (2–5). Presently, licensed or tested adjuvants can be classified into two groups: 1) immunomodulatory molecules that directly activate innate immune receptors including Toll-like receptors (TLRs), such as poly(I:C) and monophosphoryl lipid (MPL), and 2) particulate formulation systems that promote antigen delivery and uptake, such as aluminum salts, MF59, and liposomes (6). In addition, the combination of immunomodulating molecules and particulate formulations has been extensively investigated for various vaccines. For example, AS adjuvant series such as AS01, AS03, and AS04 are currently used in human vaccines for various infectious diseases, such as influenza, herpes zoster, and hepatitis B virus (7–9).

Among the currently licensed vaccine adjuvants, aluminum salts (alum) have been most widely used in human vaccines for >80 years. Although alum has proven to be effective and safe for humans, some limitations have been noted. Alum poorly induces cell-mediated immunity (CMI) or T cell responses (10); moreover, alum-mixed vaccines cannot be frozen and dried, limiting their long-term storage (11). Similarly, oil-in-water emulsions, such as MF59, are applied with other substances as TLR agonists to establish more potent CMI (12, 13). CMI plays a pivotal role in initiating protection against viral infections and the subsequent elimination of virus-infected cells (14). While subunit vaccines primarily activate T helper 2 (T_H_2) cells, live attenuated vaccines or subunit vaccines in combination with specific adjuvants trigger T helper 1 (T_H_1) cells that induce robust CMI (15). T_H_1 cells induce CMI by secreting interferon-gamma and activating B cells to produce immunoglobulin (Ig) G1 and IgG3 antibodies in humans (IgG2a and IgG2b in mice) (16, 17), which are closely related to the antibody-dependent cellular cytotoxicity (ADCC) function; Fcγ receptors of effector cells such as natural killer cells recognize the Fc region of IgG1 and IgG3 antibodies (IgG2a and IgG2b in mice) bound to infected cells (18, 19). Most adjuvants developed after alum have been shown to induce T_H_1 immune responses, with some inducing both T_H_1 and T_H_2 immune responses (2, 20–23), suggesting that the balanced induction of T_H_1 and T_H_2 immune responses is highly desirable for vaccine efficacy.

Various types of biomaterials such as microbial MPL (21), cholera toxin subunit B (24), plant-derived QS-21 (23) and synthetic CpG (22) are used as adjuvants to induce robust CMI. The saponin QS-21, a triterpinoid material isolated from *Quillaja saponaria* tree bark, induces balanced T_H_1/T_H_2 immune responses and is used as an adjuvant for the varicella zoster virus vaccine (8) and coronavirus disease 2019 (COVID-19) vaccine (25, 26). However, due to adverse side effects, such as hemolysis and pain at the injection site, as well as limited supply, practical alternatives are needed (27–29).

**Figure 1:**
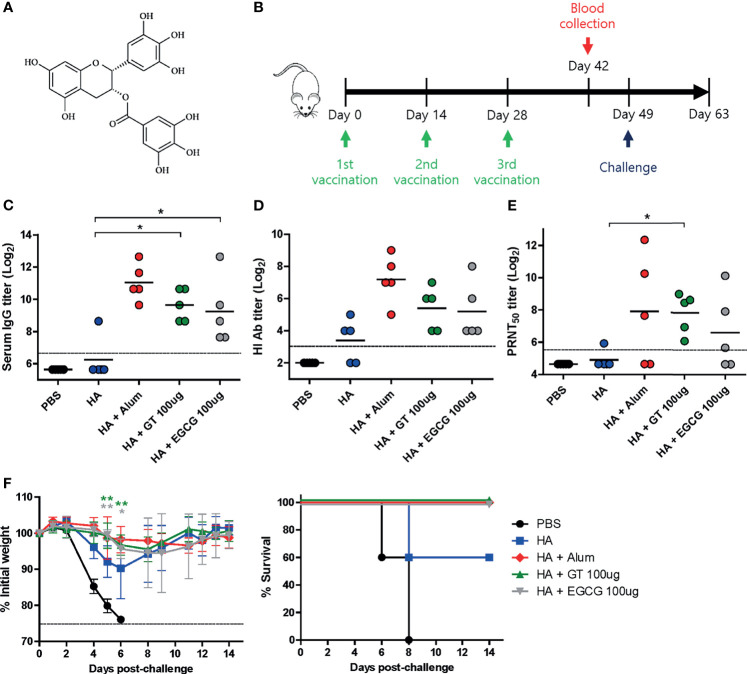
Adjuvant effects of GT or EGCG in combination with HA antigen. PBS or 4 μg of HA antigens (recombinant, full-length) with or without adjuvant (alum 50 μL, GT 100 μg, or EGCG 100 μg) were injected into mice *via* IM route thrice every 2 weeks. The vaccinated mice were challenged with 2 × 10^3^ PFU (2 MLD_50_) of wild type PR8 virus 3 weeks after the last vaccination. **(A)** Chemical structure of EGCG. **(B)** Vaccination, blood collection, and challenge schedule. **(C)** IgG antibody response to influenza PR8 virus. The antibody titer of each sample was expressed as the endpoint dilution with an absorbance value 2× greater than that of the PBS control group. The detection limit (dashed line) was 100. **(D, E)** Neutralizing antibody responses to influenza PR8 virus. HI antibody titers **(D)** and PRNT_50_ titers **(E)** against the PR8 virus are shown. The detection limits of HI assay and PRNT (dashed lines) were 8 and 50, respectively. **(F)** Protective efficacy against influenza PR8 virus. The changes in weight and survival rates of the mice were monitored. Dashed line indicates humane endpoint which means losing 25% weight loss. Mice that lost >25% body weight were euthanized. Data of antibody titers **(C–E)** were log-transformed and expressed as means (horizontal lines) and scatter plots. Data of the changes in weight (**F**, left panel) are shown as means (points) and standard deviations (error bars). One-way ANOVA followed by Tukey’s multiple comparison test was conducted to compare three or more groups. Repeated measures two-way ANOVA followed by Bonferroni’s post-test was used to analyze weight changes for different mouse groups over time. Each group was compared with the group given the non-adjuvanted HA (**P < 0.01; *P < 0.05).

Green tea (*Camellia sinensis)* catechins are polyphenolic compounds that exhibit a variety of biological activities, including antioxidant, antitumor, and antimicrobial effects (30–32). The most abundant catechin epigallocatechin-3-gallate (EGCG) (**Figure 1A**) has been shown to demonstrate antiviral activity against a wide range of viruses through diverse mechanisms (33, 34). Structure–activity relationships revealed that the 3-galloyl group of the catechin skeleton plays an important role in virucidal function (33, 34). The potent irreversible virucidal effect of catechins was harnessed for the generation of inactivated vaccines, which are highly immunogenic and provide complete protection against lethal challenges (35). The outbreak of novel swine influenza in 2009 prompted the vaccine manufactures to consider recombinant vaccine that would be supplied in a timely manner. The present study examined whether EGCG can serve as a novel vaccine adjuvant for a subunit/split influenza vaccine. The adjuvanticity of EGCG was evaluated primarily using recombinant influenza hemagglutinin (HA) in a mouse model. In addition, immune responses elicited by EGCG-adjuvanted HA were also characterized in terms of antibody profile and antibody effector functions such as ADCC. Our study is the first report on a novel class of vaccine adjuvants that have both virucidal and adjuvant activities. With well-documented health benefits and immune stimulation, EGCG is an attractive option for the development of effective and safe vaccines for pandemic preparedness.

## Materials and Methods

### Green Tea Extract and EGCG

Green tea extract (GT) was provided by Amore-Pacific Co. (Seoul, Republic of Korea) as previously described (34). It comprised caffeine (5.48%), gallic acid (0.22%), gallocatechin (1.95%), epigallocatechin (10.22%), catechin (0.35%), EGCG (9.11%), epicatechin (2.51%), and gallocatechin gallate (0.88%), as determined by high-performance liquid chromatography (HPLC). Purified EGCG (98%, powder) was purchased from Changsha Sunfull Biotech (Changsha, China). The GT and EGCG solutions were prepared by adding distilled water to the powder.

### Cell Line, Influenza Virus, Recombinant Protein, and Antigens

Madin–Darby Canine Kidney (MDCK, ATCC CCL-34) cells were cultured in minimum essential medium (MEM, HyClone, Logan, UT, U.S.) supplemented with 10% fetal bovine serum (HyClone). Influenza A/Puerto Rico/8/34 (H1N1) (PR8), A/Seoul/Y-1/2009 (H1N1), A/Philippines/2/82 (H3N2), B/Shanghai/361/2002, and B/Brisbane/60/2008 viruses were propagated in chicken embryos and stored at −80°C until further use. Recombinant HA full-length proteins derived from PR8 were purchased from Sino Biological (Beijing, China). The recombinant HA globular domains (GDs) of PR8 were expressed and purified as described previously (36). Briefly, the proteins were fused with the mouse RNA-interaction domain (RID) of lysyl-tRNA synthetase (LysRS) for soluble expression in *Escherichia coli*. The recombinant HA stalk domains (STs) of PR8 were expressed by fusion with LysRS and purified as described previously (37). The formalin-inactivated influenza virus (FAiV) was prepared by mixing 0.1% (v/v) of formaldehyde with 2 × 10^6^ PFU of influenza virus (A/PR/8/34), and the mixtures were incubated for 24 h at room temperature (RT, approximately 20-25°C). Human papillomavirus (HPV) 16 L1 virus-like particle (VLP) antigens were fused with the human RID of LysRS for soluble expression in *E. coli*. Tetravalent seasonal influenza vaccine, Kovax Flu Tetra PF (2018) (Kovax), was purchased from Korea Vaccine Inc (Seoul, Republic of Korea). All antigens were diluted in PBS. Alum, which contains an aqueous solution of aluminum hydroxide and magnesium hydroxide, was purchased from Thermo Fisher Scientific (Imject Alum, Waltham, MA, U.S.).

### Animal Experiments

All animal studies were reviewed and approved by the Institutional Animal Care and Use Committee (IACUC) of the International Vaccine Institute (IVI, Seoul, Republic of Korea) (IACUC PN 2017-019, IACUC PN 2018-008, and IACUC PN 2019-007) and carried out in the ABSL-2 facility of IVI. Six-week-old female BALB/c mice (Orient Bio, Seongnam, Republic of Korea) were vaccinated with antigens with various additives *via* intramuscular (IM, 100 μL) or intraperitoneal (IP, 200 μL) routes. Only FAiV with or without EGCG was injected *via* IP route. Blood was collected from vaccinated mice by retro-orbital bleeding under anesthesia. After vaccination, the mice were challenged by intranasal infection with the PR8 virus under anesthesia. Mice that lost >25% of body weight were considered nonviable and euthanized.

### Enzyme-Linked Immunosorbent Assay (ELISA) for Antibody (IgG, IgG1, and IgG2a) Titers

ELISA was performed to estimate the HA-specific antibodies in mouse sera. Ninety-six-well plates were coated with 10^5^ PFU of PR8 viruses or 0.05 μg of HA GD or ST proteins per well. The plates were incubated with two-fold serially diluted sera for 1 h at RT after blocking. After washing, the plates were incubated with Horseradish peroxidase (HRP)-conjugated antimouse IgG, IgG1, or IgG2a antibodies for 1 h at RT. After washing, the plates were incubated with Tetramethylbenzidine (TMB) solution for 30 min at RT. The reaction was stopped by adding 2N H_2_SO_4_ solution, and the optical density was measured at 450 nm using an ELISA reader.

### Hemagglutination Inhibition Assay and Plaque Reduction Neutralization Test

Hemagglutination inhibition (HI) assay and plaque reduction neutralization test (PRNT) were performed to measure the neutralizing antibody titers. For HI assay, sera were pretreated with a receptor-destroying enzyme overnight at 37°C and subsequently heat-inactivated for 30 min at 56°C. Next, two-fold serially diluted sera were incubated with the same volume (25 μL) of PR8 virus (4 HAU) for 1 h at 37°C. Fifty microliters of chicken red blood cells were added and incubated for 1 h at 4°C. HI antibody titers were expressed as the highest serum dilution factor that completely inhibited hemagglutination. For PRNT, sera were heat-inactivated for 30 min at 56°C, and two-fold serially diluted sera were incubated with 100 PFU of PR8 viruses for 90 min at 37°C. The mixtures were subjected to plaque assay on MDCK cells, and cells were incubated at 37°C in a 5% CO_2_ incubator until plaques became visible. PRNT_50_ titers were expressed as the serum dilution factor that resulted in 50% plaque reduction compared to the control.

### ADCC Assay

ADCC activity in mouse sera was estimated using the ADCC assay kit (mFCγRIV ADCC Reporter Bioassay, Promega, Madison, WI, U.S.). MDCK cells cultured on 96-well white plates were incubated with 10^4^ PFU of PR8 virus for 45 min at 37°C. After washing, the plates were incubated at 37°C in a 5% CO_2_ incubator overnight with trypsin-supplemented MEM. After washing, diluted mouse sera and effector cells (genetically engineered Jurkat T cell line that expresses mouse FCγRIV receptor and a luciferase reporter driven by an NFAT response element) were added to the infected cells, and the plates were incubated at 37°C in a 5% CO_2_ incubator for 6 h. Subsequently, luciferase reagent was added, and relative luminescence units (RLU) were detected using a spectrophotometer. Fold induction was calculated using the following formula: fold induction = RLU (induced − background)/RLU (no antibody control − background).

### Statistical Analysis

All experiments were conducted once, except for the purpose of the evaluation of the statistical significance. All data are expressed as means and scatter plots or error bars. Error bars indicate standard deviations (SD). Student’s *t*-test was used to compare two different groups. One-way ANOVA followed by Tukey’s multiple comparison test was conducted to compare three or more groups. Repeated measures two-way ANOVA followed by Bonferroni’s post-test was used to analyze weight changes for different mouse groups over time. Survival rate data were analyzed by log-rank (Mantel-Cox) test, but they were not statistically significant. Differences were considered statistically significant when *P* was <0.05 (***P < 0.001; **P < 0.01; *P < 0.05).

## Results

### Adjuvant Effects of GT or EGCG in Combination With HA Antigen

To examine the adjuvant effect of catechins, 4 μg of recombinant full-length HA was coadministered with GT or EGCG (**Figure 1A**) into mice, and the immunogenicity and protective efficacy of the vaccine were evaluated. Alum-adjuvanted HA was used as a control. The vaccination was repeated thrice with an interval of 2 weeks (**Figure 1B**). Sera were collected from the mice two weeks after the last vaccination to measure serum antibody titers. Mice administered HA without any adjuvant poorly induced serum IgG antibodies, and only one out of five mice generated detectable antibodies (**Figure 1C**). In contrast, robust antibodies were induced by alum, GT, and EGCG. While alum-adjuvanted HA induced the highest ELISA antibody titer of 2111, GT- and EGCG-adjuvanted HA generated antibody titers of 800 and 606, respectively. Viral HA inhibitory antibodies were considerably increased by the use of alum, GT, or EGCG compared with non-adjuvanted HA (**Figure 1D**). Alum and GT resulted in similar levels of PRNT_50_ neutralizing antibody titers; EGCG also led to a moderate increase in neutralizing antibodies compared to non-adjuvanted HA (**Figure 1E**). These results suggest that GT and EGCG enhanced neutralizing antibody generation although their potency was slightly weaker than that of alum. To assess the protective efficacy of the vaccination, vaccinated mice were challenged with 2 MLD_50_ (mouse lethal dose 50) of wild-type PR8 virus 3 weeks after the last vaccination. This challenge was lethal to non-vaccinated mice. Vaccination with non-adjuvanted HA resulted in ~10% weight loss and provided only partial protection in mice; two out of five mice succumbed following the challenge (**Figure 1F**). Conversely, alum, GT, and EGCG caused only mild weight loss and provided complete protection against lethal challenges. Although the survival rate data were not statistically significant (analyzed by log-rank test), the weight changes showed that the mice given the GT- and EGCG-adjuvanted HA were significantly less susceptible to the challenge. Taken together, our results demonstrate that GT and EGCG exhibit strong adjuvant effects by increasing the neutralizing antibody titers and protective efficacy.

### Adjuvant Effect of GT or EGCG on HA GD Antigen

**Figure 2:**
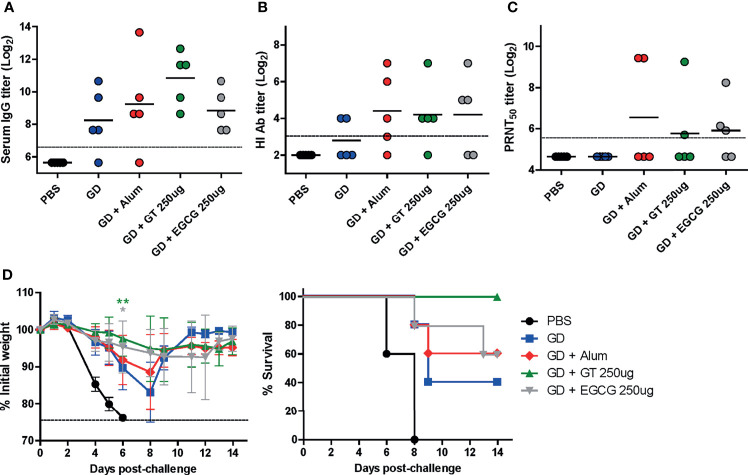
Adjuvant effect of GT or EGCG on recombinant HA GD antigen. PBS or 10 µg of GD antigens with or without alum, GT, or EGCG were injected into mice *via* IM route thrice with the interval of two weeks. The overall experimental schedule was the same as presented in **Figure 1B**. **(A)** IgG antibody response to influenza PR8 virus. The antibody titer of each sample was expressed as the endpoint dilution with an absorbance value 2× greater than that of the PBS control group. The detection limit (dashed line) was 100. **(B, C)** Neutralizing antibody responses to influenza PR8 virus. HI antibody titers **(B)** and PRNT_50_ titers **(C)** against the PR8 virus are shown. The detection limits of the HI assay and PRNT (dashed lines) were 8 and 50, respectively. **(D)** Protective efficacy against influenza PR8 virus. The vaccinated mice were challenged with 2 × 10^3^ PFU (2 MLD_50_) of wild type PR8 virus after the last vaccination, and the changes in weight and survival rates of the mice were monitored. Dashed line indicates humane endpoint which means losing 25% weight loss. Mice that lost >25% body weight were euthanized. Data of antibody titers **(A–C)** were log-transformed and expressed as means (horizontal lines) and scatter plots. Data of the changes in weight (**D**, left panel) are shown as means (points) and standard deviations (error bars). Repeated measures two-way ANOVA followed by Bonferroni’s post-test was used to analyze weight changes for different mouse groups over time. Each group was compared with the group given the non-adjuvanted GD (**P < 0.01; *P < 0.05).

The HA GD harbors the receptor-binding site and is, thus, the most important vaccine antigen for inducing neutralizing antibodies. Recombinant GD antigens, comprising the HA1 domain, were produced from *E. coli* (36) and coadministered with GT or EGCG into mice to evaluate the antibody response and protective efficacy of adjuvanted GD antigens. Compared with non-adjuvanted GD vaccine, alum-, GT-, and EGCG-adjuvanted GD vaccines induced 2, 6, and 1.5 times higher ELISA IgG antibodies, respectively (**Figure 2A**). Similar levels of HI antibody titers were observed for the three antigens, which were higher than those from the non-adjuvanted GD group (**Figure 2B**). While non-adjuvanted GD vaccine did not induce detectable PRNT_50_ neutralizing antibodies, GT- and EGCG-adjuvanted GD vaccines induced detectable PRNT_50_ neutralizing antibodies although their antibody titers were lower than that for alum-adjuvanted GD vaccine (**Figure 2C**). The vaccinated mice were challenged with 2 MLD_50_ of the wild-type PR8 virus to evaluate the protective efficacy of the vaccines. Vaccination with non-adjuvanted GD exhibited poor protection against the challenge, resulting in considerable morbidity and mortality (**Figure 2D**). Vaccination with alum- or EGCG-adjuvanted GD demonstrated a similar degree of partial protection, resulting in a survival rate of 60% following the challenge. GT-adjuvanted GD vaccine showed the most robust protection in terms of morbidity and mortality, without causing any death in mice. Overall, it appeared that the GD antigen was less immunogenic than full-length HA (**Figure 1**). Although the survival rate analyzed by log-rank test did not reach statistical significance, the weight changes showed that the mice given the GT or EGCG-adjuvanted GD were significantly less susceptible to the challenge. Thus, the combination of GT or EGCG with GD antigen improved the protective efficacy, reflected by attenuation of weight loss and mortality from lethal infection.

### Dose-Dependent Adjuvanticity of EGCG

**Figure 3:**
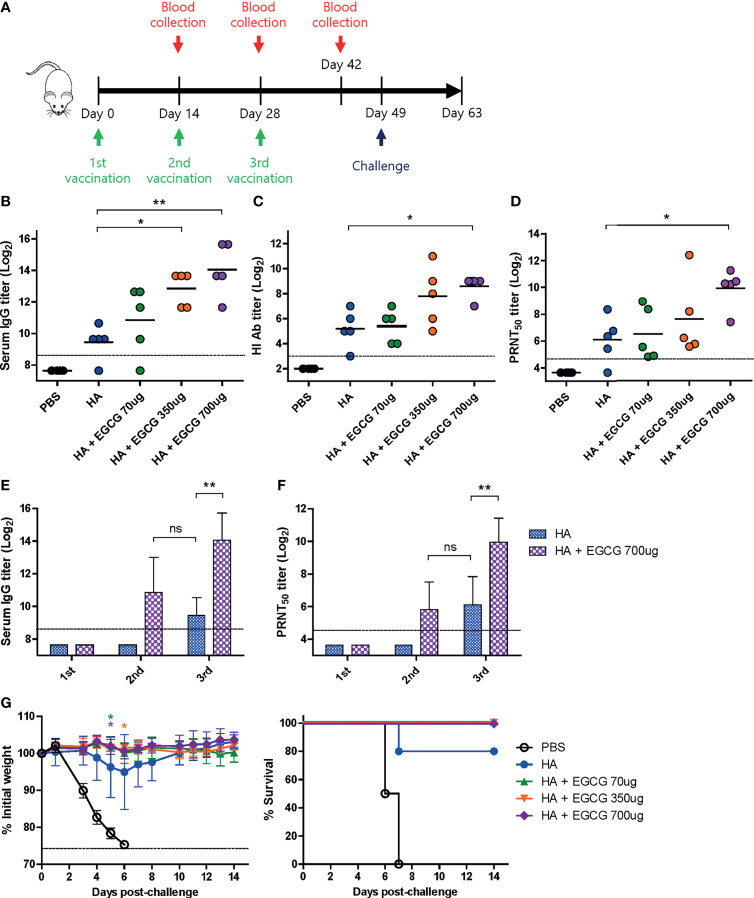
Dose-dependent adjuvanticity of EGCG. PBS or 7 µg of HA antigens with or without adjuvant (70, 350, or 700 μg of EGCG) were injected into mice *via* IM route thrice every 2 weeks. **(A)** Vaccination, blood collection, and challenge schedule. **(B)** IgG antibody response to influenza PR8 virus. The antibody titer of each sample was expressed as the endpoint dilution with an absorbance value 2× greater than that of the PBS control group. Data were log-transformed and expressed as means (horizontal lines) and scatter plots. The detection limit (dashed line) was 400. **(C, D)** Neutralizing antibody responses to influenza PR8 virus. HI antibody titers **(C)** and PRNT_50_ titers **(D)** against the PR8 virus. Data were log-transformed and expressed as means (horizontal lines) and scatter plots. The detection limits of the HI assay and PRNT (dashed lines) were 8 and 25, respectively. **(E)** IgG antibody response to influenza PR8 virus depending on the number of vaccine doses. Data were log-transformed and expressed as means (bar graphs) and standard deviations (error bars). The detection limit (dashed line) was 400. **(F)** Neutralizing antibody responses to influenza PR8 virus depending on the number of vaccine doses. Data were log-transformed and expressed as means (bar graphs) and standard deviations (error bars). The detection limit (dashed line) was 25. **(G)** Protective efficacy against influenza PR8 virus. The vaccinated mice were challenged with 4 × 10^3^ PFU (4 MLD_50_) of influenza PR8 virus after the last vaccination. Dashed line indicates humane endpoint which means losing 25% weight loss. Mice that lost >25% body weight were euthanized. Data of the changes in weight (**G**, left panel) are shown as means (points) and standard deviations (error bars). Student’s *t*-test was used to compare two different groups. One-way ANOVA followed by Tukey’s multiple comparison test was conducted to compare three or more groups. Repeated measures two-way ANOVA followed by Bonferroni’s post-test was used to analyze weight changes for different mouse groups over time. Each group was compared with the group given the non-adjuvanted HA (**P < 0.01; *P < 0.05. ns, not significant).

Based on the results that full-length HA antigens showed higher immunogenicity than GD, the dose-dependent adjuvanticity of EGCG was investigated using HA antigens as a vaccine. Three different doses of EGCG (70, 350, or 700 µg) were mixed with 7 µg of HA antigens and injected into mice thrice at the intervals of 2 weeks, and sera were collected after each vaccination to measure antibody responses (**Figure 3A**). After the third vaccination, dose dependence was clear in all criteria of immunogenicity, including ELISA, HI, and PRNT_50_ antibodies. The use of 70 µg of EGCG led to slightly higher levels of ELISA, HI, and PRNT_50_ antibodies than non-adjuvanted vaccine; however, the difference was not statistically significant (**Figures 3B–D**). Higher doses (350 µg and 700 µg) of EGCG resulted in more apparent increases in antibody titers. Compared with the non-adjuvanted vaccine, 700 µg of EGCG induced significantly higher levels of ELISA (×24), HI (×11), and PRNT_50_ (×14) antibodies, showing a clear dose-dependent increase in adjuvanticity. Serum antibody titers after each vaccination with or without 700 µg EGCG were also compared. The non-adjuvanted HA vaccine induced detectable ELISA antibodies and neutralizing antibodies only after the third vaccination (**Figures 3E, F**). In contrast, only two doses of 700 µg EGCG with HA were able to generate detectable levels of ELISA antibodies and neutralizing antibodies. Of note, the ELISA antibody titer elicited by the second dose of EGCG-adjuvanted HA vaccine was higher than that elicited by the third vaccination with the non-adjuvanted HA vaccine (**Figure 3E**). Neutralizing antibody titers were similar between the second dose of EGCG-adjuvanted HA vaccine and the third dose of non-adjuvanted HA vaccine (**Figure 3F**), suggesting robust immune-stimulating potency of EGCG. The vaccinated mice were challenged with 4 MLD_50_ of the wild-type PR8 virus to examine their protective efficacy. Mice vaccinated with non-adjuvanted HA displayed a weight loss of ~5% and were partially protected after the challenge (**Figure 3G**). However, EGCG-adjuvanted HA vaccines provided complete protection against the challenge without causing weight loss and mortality even at the lowest dose of EGCG (**Figure 3G**). Although the survival rate data were not statistically significant, the weight changes showed that EGCG, even at the lowest concentration used, significantly contributed to protective immune responses.

### Characterization of Serum IgG Antibody Subclass and ADCC Activity Elicited by EGCG-Adjuvanted HA Vaccine

**Figure 4:**
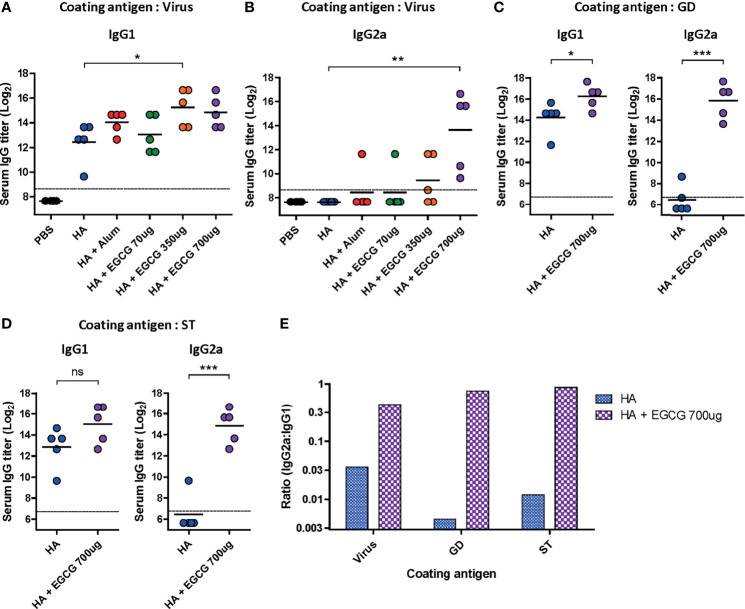
Characterization of serum IgG antibody subclass elicited by EGCG-adjuvanted HA vaccine. PBS or 7 µg of HA antigens with or without adjuvant (30 μL of alum or 70, 350, or 700 μg of EGCG) were injected into mice *via* IM route thrice every 2 weeks. Sera were collected 2 weeks after the last vaccination. **(A, B)** IgG1 **(A)** and IgG2a **(B)** antibody responses to influenza PR8 virus. The antibody titer of each sample was expressed as the endpoint dilution with an absorbance value 2× greater than that of the PBS control group. Data were log-transformed and expressed as means (horizontal lines) and scatter plots. The detection limits (dashed lines) were 400. **(C, D)** IgG1 and IgG2a antibody responses to GD **(C)** or ST **(D)** of HA protein of influenza PR8 virus. Data were log-transformed and expressed as means (horizontal lines) and scatter plots. The detection limits (dashed lines) were 100. **(E)** IgG2a/IgG1 ratios of HA and EGCG-adjuvanted HA. The bar graphs indicate geometric means. For convenience, the IgG2a/IgG1 ratios were shown as log scale; corresponding to 12 fold (virus), 190 fold (GD), and 72 fold increase (ST) by EGCG. Student’s *t*-test was used to compare two different groups. One-way ANOVA followed by Tukey’s multiple comparison test was conducted to compare three or more groups (***P < 0.001; **P < 0.01; *P < 0.05. ns, not significant).

Murine IgG antibodies are divided into four subclasses based on the differences in their structures and functions: IgG1, IgG2a, IgG2b, and IgG3 (38). Importantly, it has been shown that IgG2a has the highest affinity toward Fcγ receptors on the surface of effector immune cells; thus, it activates antibody effector functions (18, 39, 40). IgG1 and IgG2a antibody titers induced by alum- or EGCG-adjuvanted HA vaccine were compared by ELISA. While vaccination with non-adjuvanted HA induced high levels of IgG1 antibodies, the addition of alum or EGCG to the vaccine further increased the level of IgG1 antibodies (4-6 fold). (**Figure 4A**). However, no detectable IgG2a antibodies were induced by the non-adjuvanted or alum-adjuvanted HA vaccine (**Figure 4B**). Notably, EGCG dramatically increased IgG2a antibodies in a dose-dependent manner, producing >37× higher antibody titers (at 700 ug dose) than alum. Similarly, potent induction of IgG2a antibodies by EGCG was also observed when recombinant HA GD (**Figure 4C** **)** or HA ST (stalk domain mostly comprising HA2 domain) (**Figure 4D** **)** was used as a coating antigen. Remarkably, while the increase in IgG1 level was only marginal (4 fold), the increase in IgG2a level was approximately >300–700 fold in EGCG-adjuvanted HA vaccine (**Figures 4C, D**). These results show that the EGCG-adjuvanted HA vaccine induced balanced IgG2a/IgG1 antibodies (IgG2a/IgG1 ratio = approximately 0.44–0.87), whereas the non-adjuvanted HA vaccine displayed only a skewed value of IgG2a/IgG1 ratio, approximately 0.004–0.036 (**Figure 4E**).

**Figure 5:**
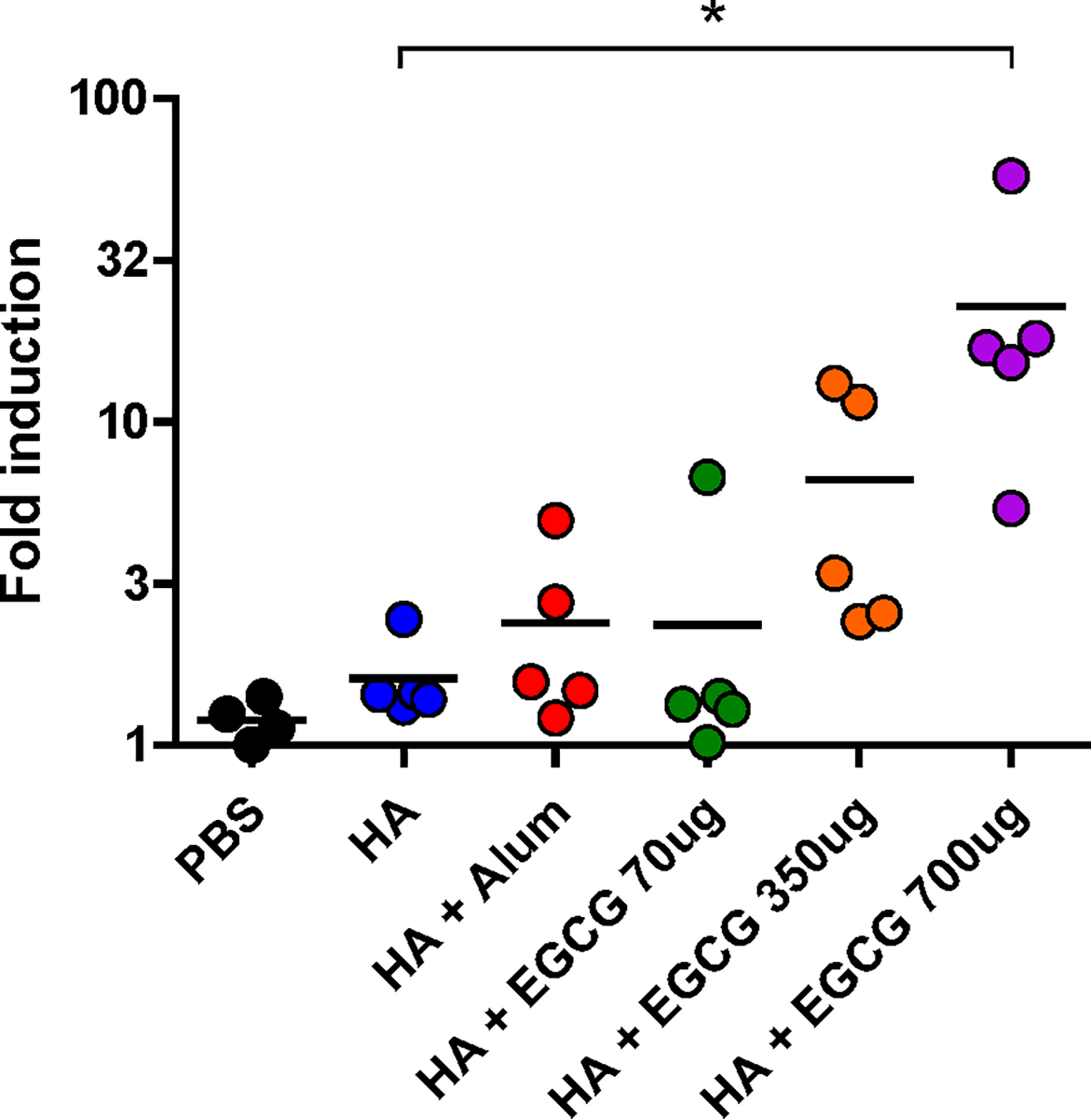
ADCC activity induced by EGCG against PR8 virus. PBS or 7 µg of HA antigens with or without adjuvant (30 μL of alum or 70, 350, or 700 μg of EGCG) were injected into mice thrice every 2 weeks. Sera were collected 2 weeks after the last vaccination. The sera were diluted 100× for the ADCC assay. Fold induction was defined as the increase in responses (number of times) compared to the negative control (reaction without mouse serum). Data are expressed as means (horizontal lines) and scatter plots. One-way ANOVA followed by Tukey’s multiple comparison test was conducted to compare three or more groups (*P < 0.05).

To examine whether antibodies induced by the vaccines have antibody effector functions, an ADCC assay was performed on the sera. Compared with non-adjuvanted HA vaccine which poorly induced ADCC activities, alum-adjuvanted HA vaccine led to a moderate increase in ADCC activity; however, this increase was not statistically significant (**Figure 5**). Furthermore, 70 µg of EGCG resulted in similar ADCC activities to alum and 350 µg of EGCG induced approximately 4 fold higher ADCC activities but did not show statistical significance. The strong induction of ADCC effector function was observed upon administration of 700 µg of EGCG-adjuvanted HA vaccine, commensurate to 14.1 and 9.5 fold increase observed for non-adjuvanted and alum-adjuvanted vaccine, respectively. A strong correlation was noted between the ADCC activities among vaccination groups and IgG2a antibody titers (**Figures 4B**, **5**), suggesting that IgG2a antibodies are primarily responsible for ADCC activity (39, 40). A dramatic isotype switching correlated with ADCC function, which is not expected in alum-adjuvanted vaccines.

### Synergistic Effect of EGCG and Alum

**Figure 6:**
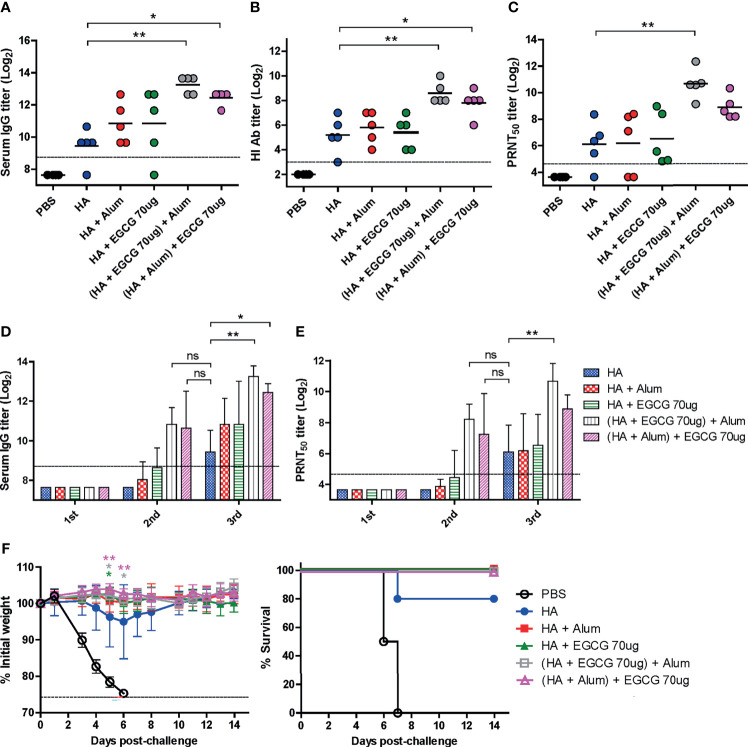
Synergistic effect of EGCG and alum. PBS or 7 µg of HA antigens with or without adjuvant (30 μL of alum, 70 μg of EGCG, or both alum and EGCG) were injected into mice thrice every 2 weeks. The overall experimental schedule was the same as presented in **Figure 3A**. **(A–C)** ELISA, HI assay, and PRNT were conducted to verify the synergistic relationship between EGCG and alum on vaccine immunogenicity. Data were log-transformed and expressed as means (horizontal lines) and scatter plots. **(A)** IgG antibody response to influenza PR8 virus. The antibody titer of each sample was expressed as the endpoint dilution with an absorbance value 2× greater than that of the PBS control group. The detection limit (dashed line) was 400. **(B, C)** Neutralizing antibody responses to influenza PR8 virus. HI antibody titers **(B)** and PRNT_50_ titers **(C)** against the PR8 virus. The detection limits of the HI assay and PRNT (dashed lines) were 8 and 25, respectively. **(D)** IgG antibody response to influenza PR8 virus depending on the number of vaccine doses. Data were log-transformed and expressed as means (bar graphs) and standard deviations (error bars). The detection limit (dashed line) was 400. **(E)** Neutralizing antibody responses to influenza PR8 virus depending on the number of vaccine doses. Data were log-transformed and expressed as means (bar graphs) and standard deviations (error bars). The detection limit (dashed line) was 25. **(F)** Protective efficacy against influenza PR8 virus. Mice were challenged with 4 × 10^3^ PFU (4 MLD_50_) of influenza PR8 virus after the last vaccination. Dashed line indicates humane endpoint which means losing 25% weight loss. Mice that lost >25% body weight were euthanized. Data of the changes in weight (**F**, left panel) are shown as means (points) and standard deviations (error bars). One-way ANOVA followed by Tukey’s multiple comparison test was conducted to compare three or more groups. Repeated measures two-way ANOVA followed by Bonferroni’s post-test was used to analyze weight changes for different mouse groups over time. Each group was compared with the group given the non-adjuvanted HA (**P < 0.01; *P < 0.05. ns, not significant).

Several adjuvants consist of two or more components as immune-stimulating materials in the particulate form (3). Therefore, we examined whether the combined use of EGCG and alum can generate synergistic effects on the immunogenicity of the HA antigen. As the order of mixing the components may affect the immunogenicity, two different methods of combining alum and EGCG with HA antigens were compared: 1) (HA + EGCG) + alum (HA proteins were first mixed with EGCG and then alum was added to the mixture) and 2) (HA + alum) + EGCG (HA proteins were first mixed with alum and then EGCG was added to the mixture). Compared with non-adjuvanted HA, alum and EGCG increased ELISA IgG antibodies, but the increase was not significant (**Figure 6A**). However, for both the formulations, a significant increase in ELISA antibodies was observed, with the first formulation inducing higher antibody titers than the second. Neutralizing HI and PRNT_50_ antibodies showed very similar patterns to ELISA antibody titers among the vaccination formulations. The combination of EGCG and alum induced higher levels of neutralizing antibodies than their individual administration, and while there was no statistical significance, the (HA + EGCG) + alum formulation consistently showed higher potency than the (HA + alum) + EGCG formulation (**Figures 6B, C**). Thus, results suggest that EGCG and alum synergistically enhance neutralizing antibody responses of HA, and the mixing order may influence the immunogenicity. Serum antibody titers after each vaccination with or without adjuvant were also compared (**Figures 6D, E**). The ELISA and neutralizing antibody titers of the second dose in combination of EGCG and alum were comparable to those of the third dose without combination, demonstrating a dose-sparing effect. The protective efficacy of all adjuvanted HA vaccines was complete without causing any morbidity following the lethal challenge in mice (**Figure 6F**), suggesting that EGCG alone or in combination with alum induced neutralizing antibodies sufficient for protection.

### Adjuvant Effect of EGCG to Other Types of Vaccine

**Figure 7:**
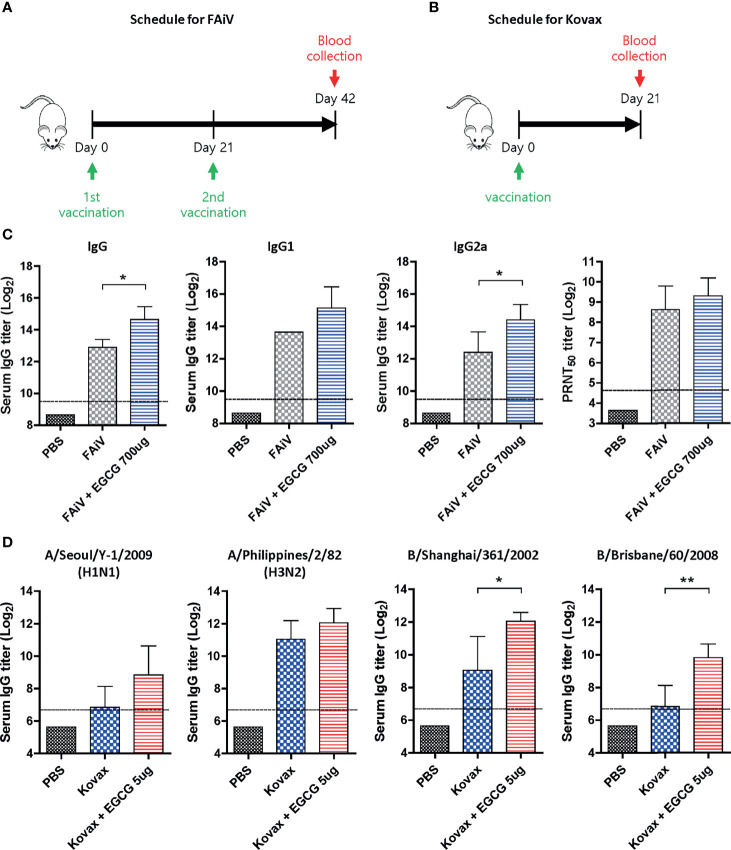
Adjuvant effect of EGCG to other types of vaccine. **(A)** Vaccination and blood collection schedule for FAiV. PBS or 2 × 10^6^ PFU of FAiV with or without EGCG were injected into mice *via* IP route twice every 3 weeks. Sera were collected 3 weeks after the last vaccination. **(B)** Vaccination and blood collection schedule for Kovax is shown. PBS or 6 μg of Kovax (1.5 μg of each antigen derived from four different strains) with or without EGCG were injected into mice *via* IM route once. Sera were collected 3 weeks after the vaccination. **(C)** Total IgG, IgG1, and IgG2a antibody responses or neutralizing antibody responses to influenza PR8 virus. The detection limit of ELISA antibody and PRNT_50_ titers (dashed lines) were 800 and 25, respectively. **(D)** Serum IgG titers specific for four different influenza strains are shown. The detection limits (dashed lines) were 100. Data of antibody titers **(C, D)** were log-transformed and expressed as means (bar graphs) and standard deviations (error bars). Student’s *t*-test was used to compare two different groups (**P < 0.01; *P < 0.05).

Given that most of the currently licensed inactivated viral vaccines are formalin-inactivated, the adjuvanticity of EGCG was investigated using FAiV. FAiV was injected into mice twice at an interval of 3 weeks with or without EGCG for comparison of antibody responses between the two groups (**Figure 7A**). Coadministration of EGCG with FAiV induced three to four times higher levels of serum total IgG, IgG1, and IgG2a antibodies than that of FAiV-only group (**Figure 7C**). Likewise, a higher level of neutralizing antibodies was induced when EGCG was coadministered with FAiV, but the difference was not statistically significant. Next, the adjuvanticity of EGCG was also tested using a commercial quadrivalent influenza split vaccine containing four antigens derived from A/Singapore/GP1908/2015 IVR-180 (H1N1), A/Singapore/INFIMH-16-0019/2016 IVR-186 (H3N2), B/Phuket/3073/2013, and B/Maryland/15/2016 NYMC BX-69A. The split vaccine with or without EGCG was injected once into mice, and sera were collected 3 weeks after vaccination to measure the antibody response (**Figure 7B**). ELISA assays using influenza viruses as coating antigens revealed generation of >2–8× higher levels of IgG antibodies against a variety of viruses, including A/Seoul/Y-1/2009 (H1N1), A/Philippines/2/82 (H3N2), B/Shanghai/361/2002 (B/Yamagata lineage), and B/Brisbane/60/2008 (B/Victoria lineage) strains (**Figure 7D**). These results clearly indicate that EGCG can improve the immunogenicity of inactivated viral vaccine and seasonal quadrivalent split vaccine in addition to the recombinant protein vaccine (**Figures 1**, **3**), demonstrating the versatility of EGCG as a novel vaccine adjuvant.

## Discussion

This study presents EGCG as a novel vaccine adjuvant for viral vaccines. Although GT also had an adjuvant effect as shown in **Figures 1**, **2**, it was not further pursued in the present study, considering potential variations of its chemical composition, which could pose difficulties for quality control. The effect of EGCG was proven for both recombinant HA [full-length or GD comprising receptor-binding domain from bacterial host (36, 41)] and virus-derived HA (inactivated split vaccine). HA antigens without adjuvants poorly induced neutralizing antibodies and provided only partial protection against lethal challenge in mice. In contrast, the coadministration of EGCG with HA antigens resulted in robust virus-specific ELISA responses and neutralizing antibodies, providing complete protection against the challenge. Recombinant protein antigens are usually poorly immunogenic because of their small size. The oligomeric assembly of monomeric antigens in a highly repetitive manner (as VLPs or nanoparticles) is the key to activate B-cells (42–44). Thus, particulate formation and/or adjuvant usage with antigens is crucial for improving the immunogenicity of subunit vaccines (45). EGCG has been shown to induce protein multimerization (46, 47) and cross-link with viral antigens mainly through sulfhydryl linkages (35). EGCG is an aromatic compound comprising a large number of hydroxyl groups with the ability to interact with protein antigens *via* multiple hydrogen bonds, aromatic stacking, and hydrophobic interactions. It is possible that the EGCG-induced multimerization of monomeric antigens into larger particulate forms may enable their uptake by antigen-presenting cells (45, 46). In addition to multimerization, there may be some other effects that improve the immunogenicity of vaccines. The molecular mechanisms underlying the immune-stimulating activity of EGCG other than antigen multimerization remain unclear although naturally observed EGCG derivatives have recently been shown to activate TLR-5 (48). It should also be mentioned that while EGCG was able to improve the immunogenicity of recombinant proteins and inactivated vaccines, it either slightly affected or reduced the antibody response of HPV VLPs (**Supplementary Figure S1**), probably due to interfering with the physical integrity of VLPs. It is likely that the same type of interactions (hydrophobic or H-bond) between EGCG and the target protein may have a beneficial effect by increasing the immunogenicity of monomeric soluble antigens by their multimerization or prove detrimental for VLP vaccines by interfering with the regular assembly of viral antigens. Thus, a judicious choice of vaccine type and compatibility of the vaccine manufacturing process is required for the best application of EGCG as a vaccine adjuvant.

Antibody effector functions such as ADCC, antibody-dependent cellular phagocytosis, and complement-dependent cytotoxicity provide additional correlates of protection in addition to direct neutralization by antibodies, improving the potency and breadth of protection (49–52). It has been shown that the induction of antibody effector functions by adjuvants translates into improved vaccine efficacy (53, 54). In particular, antibody effector functions have emerged as critical correlates of protection for the development of broadly protective universal influenza vaccines (55–57). Our data suggest that EGCG is a novel vaccine adjuvant that can induce ADCC-mediating antibodies. While alum induced an antibody response dominated by IgG1 antibodies, EGCG robustly induced IgG2a as well as IgG1 antibodies that correlated with ADCC activity (**Figures 4A, B**, **5**). These results are consistent with the more potent character of IgG2a for the FcγR-mediated effector functions (39, 40, 58). It would be worthwhile to examine whether EGCG-exerted effector function would impart broader protection against heterologous influenza viruses through further studies (56, 57). In mice, IgG1 and IgG2a antibody responses represent T_H_2 and T_H_1 immune responses, respectively (59). Considering that the balanced induction of T_H_1/T_H_2 immune response is a highly desirable trait for the development of novel vaccine adjuvants (2, 60), EGCG provides an option for the induction of antibody-mediated neutralizing and effector functions.

Our data demonstrates a synergistic effect between EGCG and alum. A significant increase in neutralizing antibody levels was observed when HA antigens were combined with both alum and EGCG (**Figure 6**). Notably, the order in which alum and EGCG were added to the HA antigens influenced the neutralizing antibody titers. We believe that the multimerization of HA proteins by EGCG should precede its adsorption onto alum for optimal immunogenicity of recombinant protein antigens. The synergistic effects of EGCG and alum strongly suggest that EGCG can be compatibly used with alum-adjuvanted currently licensed vaccines for further improvement of vaccine efficacy. For example, the use of AS04 adjuvant comprising alum and MPL in Cervarix, a licensed HPV vaccine, induced robust immune responses (61). Dose-sparing is an important issue in increasing the global supply of vaccines, especially in the case of a pandemic. Thus, the present results are directly relevant to the immediate supply of pandemic influenza vaccines (62, 63). The ongoing COVID-19 pandemic underscores the need for rapid supply of vaccines. The dose-sparing effect of adjuvants could increase the volume of pandemic vaccines in a timely manner. As a representative plant-derived adjuvant, QS-21 is in high demand for the COVID-19 vaccine (25, 26). However, this triterpenoid moiety requires extensive fractionation from high molecular weight saponins with various modifications. In contrast, the catechin EGCG (molecular weight 458 Da) in high purity is easy to supply, making it a suitable, novel biomaterial adjuvant for pandemic vaccines.

While EGCG presents as an option for developing safe and effective vaccines, some issues remain to be addressed for the clinical and practical application of EGCG as a vaccine adjuvant. The present evaluation based on a mouse model should be preferably extended to a ferret challenge with a wide spectrum of antigen dose. Although most of data in this study showed that EGCG-adjuvanted HA were significantly superior to non-adjuvanted HA, the difference in survival rate was not statistically significant presumably by using a very high concentration of HA protein as a comparator, and we plan to address it in the future by repeating the experiments with lower antigen dose. EGCG interferes with the single radial immunodiffusion or ELISA-based assays for quantitative estimation of vaccine antigens (data not shown), requiring an alternative potency assay for the licensure of EGCG-adjuvanted vaccine (64, 65). Moreover, the safety and tolerability of EGCG should be evaluated in the context of vaccine delivery although safety and various types of health benefits of GT as a beverage have been documented. The induction of both humoral immunity and CMI is essential for improved protection. While the present study successfully demonstrated a robust induction of neutralizing antibodies and antibody effector function by EGCG, further studies are required to investigate the molecular mechanisms of adjuvant function and potential cross-protection. Additionally, as reflected in the IgG2a/IgG1 ratio, the choice of vaccine type needs to be screened for the best application of EGCG as a vaccine adjuvant with respect to modulating antibody effector function. It is also necessary to evaluate the adjuvant effect of EGCG against other vaccine antigens for broader applications. However, the potential of EGCG to improve the immunogenicity and protective efficacy of currently licensed vaccines warrants further investigation. Although this study mainly focuses on using EGCG as an adjuvant, Potent virucidal effects of GT catechins have been previously documented for a variety of viruses (34, 66). Thus, the dual function of EGCG, virucidal and adjuvanting, could be used to improve inactivated whole virus/split vaccine as well as for recombinant/subunit vaccines.

In conclusion, this study presents EGCG as a novel adjuvant for subunit, split, and inactivated vaccines. In addition to robust neutralizing antibodies, EGCG induced ADCC-mediating antibodies and balanced T_H_1/T_H_2 immunity. As a plant-derived biomaterial, EGCG catechin can serve as a novel adjuvant for the development of safe and effective vaccines with increased public acceptance. The dose-sparing effect of stand-alone or combination of adjuvants will also address vaccine supply issues in pandemic outbreaks.

## Data Availability Statement

The original contributions presented in the study are included in the article/**Supplementary Material**. Further inquiries can be directed to the corresponding authors.

## Ethics Statement

The animal study was reviewed and approved by Institutional Animal Care and Use Committee (IACUC) of the International Vaccine Institute (IVI, Seoul, Republic of Korea).

## Author Contributions

YC and BS designed the study. YC, JA, SB, YJ, and BS analyzed the data and wrote the manuscript. YC performed overall experiments. MK, HO, JL, and WC performed animal experiments. SY, MSK, and JY provided recombinant protein antigens and technical assistance. All authors contributed to the article and approved the submitted version.

## Funding

This work was supported in part by Brain Korea 21 (BK21) FOUR program and National Research Foundation of Korea (NRF) grant funded by the Korean government (Ministry of Science, ICT and Future Planning), grant number 2020R1A2C1010703.

## Conflict of Interest

The authors declare that the research was conducted in the absence of any commercial or financial relationships that could be construed as a potential conflict of interest.

## Publisher’s Note

All claims expressed in this article are solely those of the authors and do not necessarily represent those of their affiliated organizations, or those of the publisher, the editors and the reviewers. Any product that may be evaluated in this article, or claim that may be made by its manufacturer, is not guaranteed or endorsed by the publisher.
