# Supplementary material for: Epigallocatechin-3-Gallate as a Novel Vaccine Adjuvant
Source: Front Immunol. 2021 Nov 12;12:769088. doi: 10.3389/fimmu.2021.769088 (PMC8632720; doi:10.3389/fimmu.2021.769088)
Supplement: Supplementary file 1 [file DataSheet_1.docx]

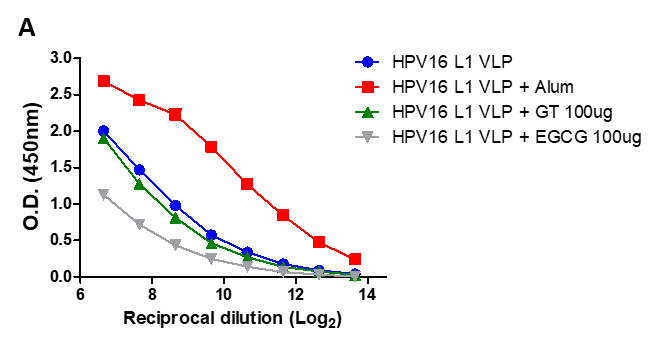


**Supplementary Figure 1.** **Adjuvant effects of GT or EGCG to HPV16 L1 VLP antigen.** Four micrograms of the antigens (produced in *E.coli*) with or without adjuvant (Alum 50 μL, GT 100 μg, or EGCG 100 μg) were injected into mice via IM route thrice every 2 weeks. Sera were collected 2 weeks after the last vaccination. IgG antibody response to HPV16 L1 VLP (Abcam, Cambridge, UK) is shown. Two-fold serially diluted pooled sera in each group were tested thrice.
